# Major adverse cardiovascular events with basal insulin peglispro versus comparator insulins in patients with type 1 or type 2 diabetes: a meta-analysis

**DOI:** 10.1186/s12933-016-0393-6

**Published:** 2016-05-17

**Authors:** Byron J. Hoogwerf, A. Michael Lincoff, Angel Rodriguez, Lei Chen, Yongming Qu

**Affiliations:** Lilly Corporate Center, Eli Lilly and Company, Drop Code 2240, Indianapolis, IN 46285 USA; Cleveland Clinic Coordinating Center for Clinical Research (C5 Research), Cleveland Clinic, Cleveland, OH USA; Lilly Spain, Eli Lilly and Company, Alcobendas, Spain

**Keywords:** Diabetes, Cardiovascular disease, Insulin

## Abstract

**Background:**

To identify possible differences in cardiovascular (CV) risk among different insulin therapies, we performed pre-specified meta-analyses across the clinical program for basal insulin peglispro (BIL), in patients randomized to treatment with BIL or comparator insulin [glargine (IG) or NPH].

**Methods:**

One phase 2 (12-week) and 6 phase 3 (26 to 78-week) randomized studies of BIL compared to IG or NPH, in patients with type 1 or type 2 diabetes, were included. The participants were diverse with respect to demographics, baseline glycemic control, and concomitant disease or medications, but treatment groups were comparable in each study. For any potential CV or neurovascular event, relevant medical information was provided to a blinded external clinical events committee (C5Research, Cleveland Clinic, Cleveland, OH, USA) for adjudication. Cox regression analysis was used to compare treatment groups. The primary endpoint was a composite of adjudicated MACE+ [CV death, myocardial infarction (MI), stroke, or hospitalization for unstable angina].

**Results:**

The pooled population included 5862 patients in the safety evaluation, with randomization to BIL:IG:NPH of 3578:2072:212. Mean age was 54.1 years, 27 % had type 1 diabetes, 56 % were male, and 88 % were white. Baseline demographic and clinical characteristics, including use of statins or other lipid-lowering drugs, were comparable between BIL and comparators. A total of 83 patients experienced at least 1 MACE+ and 70 patients experienced at least 1 MACE (CV death, MI, or stroke). Overall, there were no treatment-associated differences in time to MACE+ [hazard ratio (HR) for BIL versus comparator insulin (95 % CI): 0.82 (0.53–1.27)] or MACE [0.83 (0.51–1.33)]. In 4297 patients with type 2 diabetes, there were 71 MACE+ events [HR: 1.02 (95 % CI: 0.63–1.65), p = 0.94]. In 1565 patients with type 1 diabetes, there were only 12 MACE+ [0.24 (0.07–0.85), p = 0.027]. There were no differences in all-cause death between BIL and comparators. Sub-group analyses did not identify any sub-population with increased risk with BIL versus comparator insulins.

**Conclusions:**

Treatment with BIL versus comparator insulin in patients with type 1 diabetes or type 2 diabetes was not associated with increased risk for major CV events in the studies analyzed.

**Electronic supplementary material:**

The online version of this article (doi:10.1186/s12933-016-0393-6) contains supplementary material, which is available to authorized users.

## Background

Diabetes mellitus is associated with an increased risk for cardiovascular disease (CVD). Both observational data and intervention trials support the concept that dyslipidemia, hypertension, and thrombogenic variables likely contribute to CVD risk in type 2 diabetes [1, 2]. Increasing duration of diabetes is associated with increased CVD risk, as well as a likely need for insulin therapy. However, the effect of insulin use on CVD risk is unclear. In the ORIGIN trial, insulin glargine use versus standard of care in patients with impaired glucose tolerance or early diabetes was not associated with any CVD benefit or risk [3]. The UKPDS, a study in patients newly diagnosed with type 2 diabetes, suggested a CVD benefit in those individuals randomized to an intensive policy using oral glucose-lowering medications or insulin, compared to those who received the conventional policy [4–6]. Studies in patients with type 2 diabetes with more advanced disease have not shown benefits of intensive glucose control to reduce CVD risk [7–9]. In type 1 diabetes, the single large intervention trial (DCCT) and post-trial follow up (EDIC) compared intensive versus conventional insulin therapy and showed a reduction in CVD with intensive glycemic treatment in the long-term follow-up study [10, 11].

By contrast, there are studies that suggest that insulin therapy may be associated with increased CVD risk. Hypoglycemia was associated with increased mortality risk in the ACCORD trial [12], and a companion report by Miller and colleagues [13] reported that in patients on any insulin, the risk for hypoglycemia requiring the help of another person increased by more than fourfold. Siraj and associates used the ACCORD data to analyze whether insulin dose was associated with increased cardiovascular mortality [14]. In univariate analyses there was an association, but this association was no longer statistically significant after adjustment for baseline covariates. Whereas the mechanism by which insulin may contribute to CVD risk or mortality is unclear, several investigators have shown that insulin-associated hypoglycemia is also associated with cardiac arrhythmias [15–18]. Comparisons among insulin analogs that may have greater efficacy or reduced risk for hypoglycemia are limited. Only recently was a meta-analysis for CV events performed in the phase 3 insulin degludec trials versus other insulins. In this meta-analysis there were 80 MACE+ [CV death, nonfatal myocardial infarction (MI), nonfatal stroke, and unstable angina with hospitalization] with a HR 1.097 (95 % CI of 0.681, 1.768), and 54 MACE (MI, stroke, CV death) with a HR 1.393 (95 % CI 0.757–2.565) [19]. The current report extends the data on CVD events with different insulin analogs.

Basal insulin peglispro (BIL) is a novel basal insulin with a large hydrodynamic size [20]. In phase 1 studies, BIL was shown to have slower absorption from the site of injection, slower clearance from the circulation, and a prolonged duration of action compared to conventional basal insulin [21, 22]. In addition, BIL was shown to have reduced peripheral activity, resulting in hepato-preferential action [23, 24].

In order to identify possible differences in CV risk among different insulin therapies, pre-specified meta-analyses for CVD events were performed across the clinical program using an integrated database of a phase 2 study and 6 phase 3 studies with active comparators. The primary outcome variable was the composite of major adverse CV events (MACE+) based on independent adjudication. Secondary outcome endpoints included the composite of adjudicated MACE, as well as each individual component of adjudicated MACE+, and all-cause death.

## Methods

### Studies and patients

The phase 3 program for BIL included six studies with an active comparator; two studies in type 1 diabetes (N = 1569, all randomized patients; 1565 in the safety evaluation) and 4 studies in type 2 diabetes (N = 4014, all randomized patients; 4009 in the safety evaluation), with three studies on basal insulin only and one study on basal-bolus insulin [25–30]. Three of the studies were double-blind [26–28]. The integrated safety analyses included these 6 phase 3 studies and 1 phase 2 study in type 2 diabetes with an active comparator (N = 289, all randomized patients, 288 in the safety evaluation) [31], each of which had a pre-defined CV event adjudication process. Adjudication was not performed in a phase 2 Study in patients with type 1 diabetes since that study was completed prior to FDA request for adjudication of CV events in patients with type 1 diabetes. All of the studies in the meta-analysis were conducted in accordance with the International Conference on Harmonization Guidelines for Good Clinical Practice and the Declaration of Helsinki. All patients signed an informed consent document, and the protocols and consent documents were approved by local ethical review boards prior to study initiation. The studies were registered at clinicaltrials.gov as follows: NCT01435616, NCT01468987, NCT01481779, NCT01454284, NCT01582451, NCT01790438, NCT01027871.

In the BIL clinical trials, potential CV events were identified through several different approaches including investigator-identified, customized strategy based on the Medical Dictionary for Regulatory Activities (MedDRA) search terms, or based on review of adjudication package or severe adverse events/adverse events. If a potential event was identified by any of these approaches, a request to provide specific information was sent to the site and any materials related to a possible event, such as the relevant clinical details and associated laboratory tests, electrocardiograms, and imaging studies, were forwarded to the CEC (C5Research, Cleveland Clinic, Cleveland, OH, USA) for adjudication. The CEC blindly adjudicated the events based on a pre-specified event definition and rendered an assessment as to whether the case represented a confirmed event, a non-event [for example, not MACE+ or transient ischemic attack (TIA)] or lacked sufficient documentation for confirmation of an event. All TIAs were adjudicated to insure that no stroke event was missed.

### Statistical analysis

The primary outcome of MACE+ was analyzed using a Cox proportional hazard model with independent variables of treatment group (BIL versus comparator insulins) and type of study population (type 1 diabetes, type 2 diabetes insulin naïve, and type 2 diabetes previously treated with insulin), with Firth bias correction [32]. The Kaplan–Meier curve for the cumulative probability of events was estimated for each treatment group. Similar analyses were conducted for the secondary CV endpoints of MACE and individual MACE+ components, as well as all-cause death. As supportive analyses, the treatment incidence rate (per 100 patient-years) was compared between treatments using the Mantel–Haenszel test stratified by study population.

To examine if the treatment effect was different across certain risk factors, subgroup analyses were performed using a Cox regression model with independent variables of treatment group, subgroup and treatment-by-subgroup interaction for the following variables: type of diabetes, sex, age (>65, ≤65 years), disease duration (>10, ≤10 years), prior history of CVD, prior history of hypertension, lipid-lowing medication use, BMI (≥30, <30 kg/m^2^), and race (white, not white).

Per the modified intent-to-treat principle, the analysis period was from first dose of study drug to end of follow-up; any events captured from first dose of study drug to end of study (including the 4-week follow up period, as well as the period after early termination of study drug but staying in the study) were included in the CV risk analysis. Time to first event was calculated from the date of first dose of the study drug to the date of first event occurrence. Patients who discontinued the study early or completed the study without developing events were right-censored at the date of the last visit.

## Results

A total of 5862 patients from seven clinical trials were included in the meta-analysis, 3578 who were randomized to treatment with BIL, 2072 to IG, and 212 to NPH. Baseline demographic and clinical characteristics of the overall patient population is given in Table 1; approximately 27 % had type 1 diabetes, mean diabetes duration was 14 years, and 88 % were white. There were slightly more men in the comparator insulin group.

**Table 1:** Patient demographic and baseline characteristics

	Comparator (N = 2288)^a^	BIL (N = 3584)^a^
Type 1 diabetes, n (%)	610 (26.7)	959 (26.8)
Age, years	54.2 ± 13.3	54.0 ± 13.3
Male, %	58.0	54.7*
Race, n (%)		
American Indian/Alaska native	23 (1.0)	37 (1.0)
Asian	102 (4.5)	182 (5.1)
Black/African American	120 (5.2)	182 (5.1)
Multiple	21 (0.9)	29 (0.8)
Native Hawaiian/Pacific Islander	4 (0.2)	7 (0.2)
White	2018 (88.2)	3143 (87.8)
Hispanic or Latino, n (%)	332 (14.5)	580 (16.2)
Region, n (%)		
North America	1019 (44.5)	1611 (45.0)
European Union	834 (36.5)	1282 (35.8)
Japan	42 (1.8)	70 (2.0)
Other	393 (17.2)	621 (17.3)
BMI, kg/m^2^	30.7 ± 5.7	30.6 ± 5.7
Body weight, kg	88.6 ± 19.5	87.7 ± 19.1
Duration of diabetes, years	14.2 ± 9.4	13.8 ± 9.1
HbA1c, %	8.2 ± 1.1	8.2 ± 1.1
Hypertension, n (%)	1588 (69.4)	2458 (68.6)
Triglycerides, mg/dL	135 ± 86	139 ± 93
Total cholesterol, mg/dL	176 ± 38	177 ± 39
LDL-C, mg/dL	97 ± 33	98 ± 33
HDL-C, mg/dL	52 ± 16	52 ± 16
History of myocardial infarction, n (%)	93 (4.2)	149 (4.4)
History of coronary revascularization, n (%)	83 (3.8)	121 (3.6)
History of coronary artery bypass graft, n (%)	56 (2.6)	92 (2.7)
Lipid lowering medication, n (%)	1274 (55.7)	1965 (54.8)
Statin	1165 (50.9)	1787 (49.9)
Non-statin	316 (13.8)	499 (13.9)
Smoking status, n (%)		
Never used	1279 (58.3)	2013 (59.4)
Current used	340 (15.5)	507 (15.0)
Ever used	575 (26.2)	867 (25.6)

Mean ± SD unless otherwise specified. p value >0.05 unless specified

* p = 0.031

^a^Includes all randomized patients

The studies provided 3278 patient-years of exposure to BIL and 2016 patient-years of exposure to comparator insulins in three patient groups: type 1 diabetes, type 2 diabetes on a basal-bolus regimen, and type 2 diabetes on basal insulin only. Results of the adjudication processes are summarized in the Additional file 1: Table S1. In addition to 125 investigator-reported potential events resulting in 93 adjudicated events, use of MedDRA search terms identified an additional 124 potential events, resulting in eight adjudicated events (MI: 3; unstable angina 3; stroke: 1; TIA: 1).

**Fig. 1:**
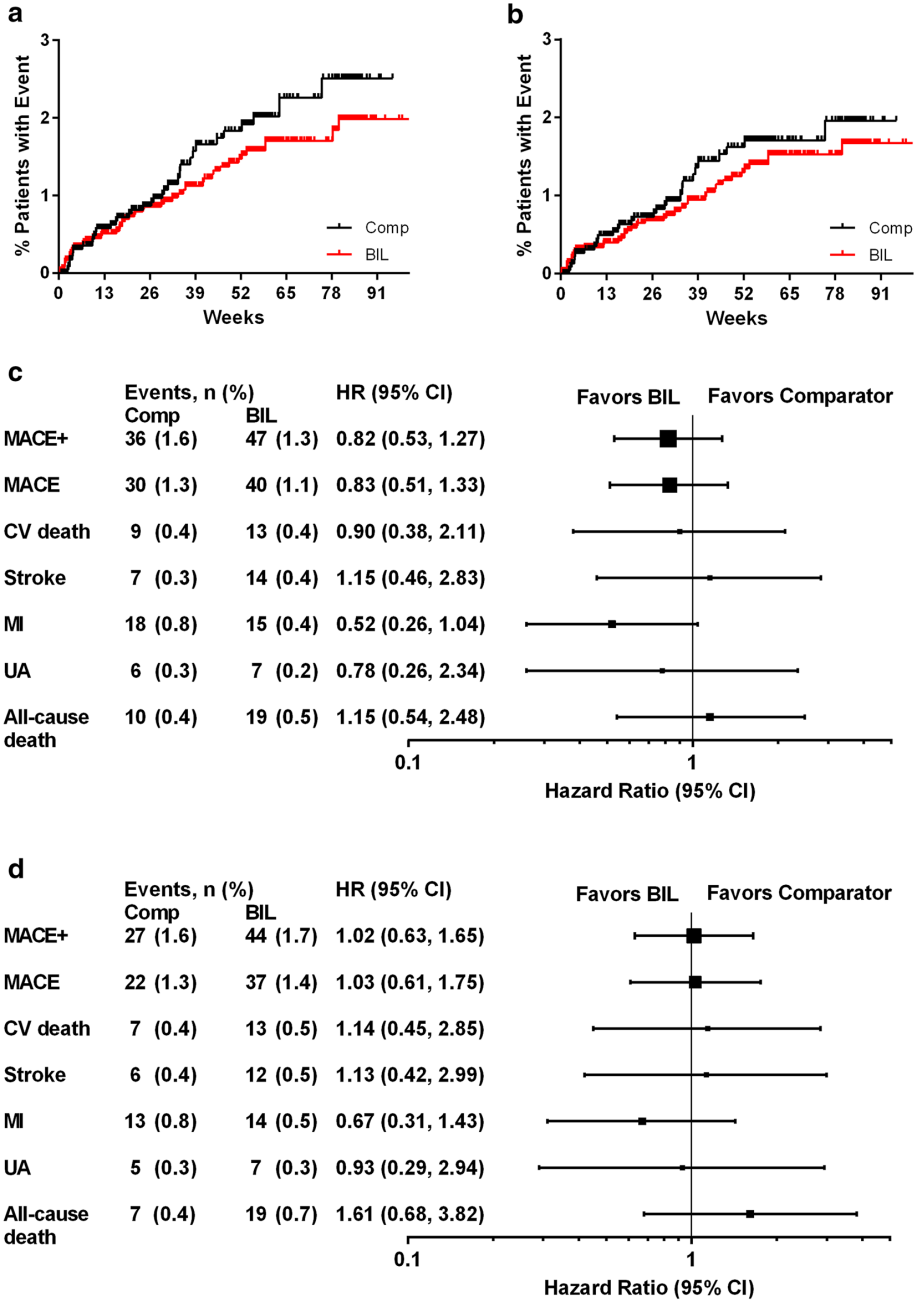
**a**, **b** Kaplan–Meier curves for time to first MACE+ and MACE, respectively, in the meta-analysis. **c**, **d** Hazard ratios (HR) and 95 % CI for risk of MACE+, MACE, components, and all-cause death for treatment with BIL versus comparator insulin in the meta-analysis (**c** all patients; **d** type 2 diabetes). *Comp* comparator insulin; *BIL* basal insulin peglispro

A total of 83 patients experienced at least one component of the MACE+ composite and 70 patients experienced at least one component of the MACE composite. The Kaplan–Meier curves for time to first MACE+ and MACE are shown in Fig. 1a and b, respectively. The overall incidence rates per 100 patient-years for MACE + were 1.8 and 1.4 for treatment with comparator insulins and BIL, respectively, and for MACE were 1.5 and 1.2 for treatment with comparator insulins and BIL. The Cox regression analysis showed that treatment with BIL versus comparator insulins in the overall patient population had a HR (95 % CI) of 0.82 (0.53–1.27) for MACE+ and 0.83 (0.51–1.33) for MACE (Fig. 1c). Event rates and HR for the individual components of MACE+ (CV death, stroke, MI, or unstable angina) and for all-cause death for BIL and comparator insulin groups are shown in Fig. 1c. In supportive analyses, the differences in incidence rates (BIL minus comparator insulin) were −0.31 (−1.02 to 0.41) events per 100 patient-years for MACE+ and −0.23 (−0.88 to 0.42) events per 100 patient-years for MACE.

The incidence rates for adjudicated MACE+ or MACE by individual study or patient group were not statistically different between BIL and comparator insulin treatment, except for patients with type 1 diabetes (Additional file 1: Table S2). The HR for MACE+ for patients with type 1 diabetes (integrated results of two studies) for treatment with BIL versus comparator insulin was 0.24 [95 % CI: 0.07–0.85], nominal p = 0.027. The HR for MACE+ for patients with type 2 diabetes (integrated results of five studies) showed no statistically significant difference in risk for patients treated with BIL versus comparator insulin [1.02 (0.63–1.65), p = 0.94].

**Fig. 2:**
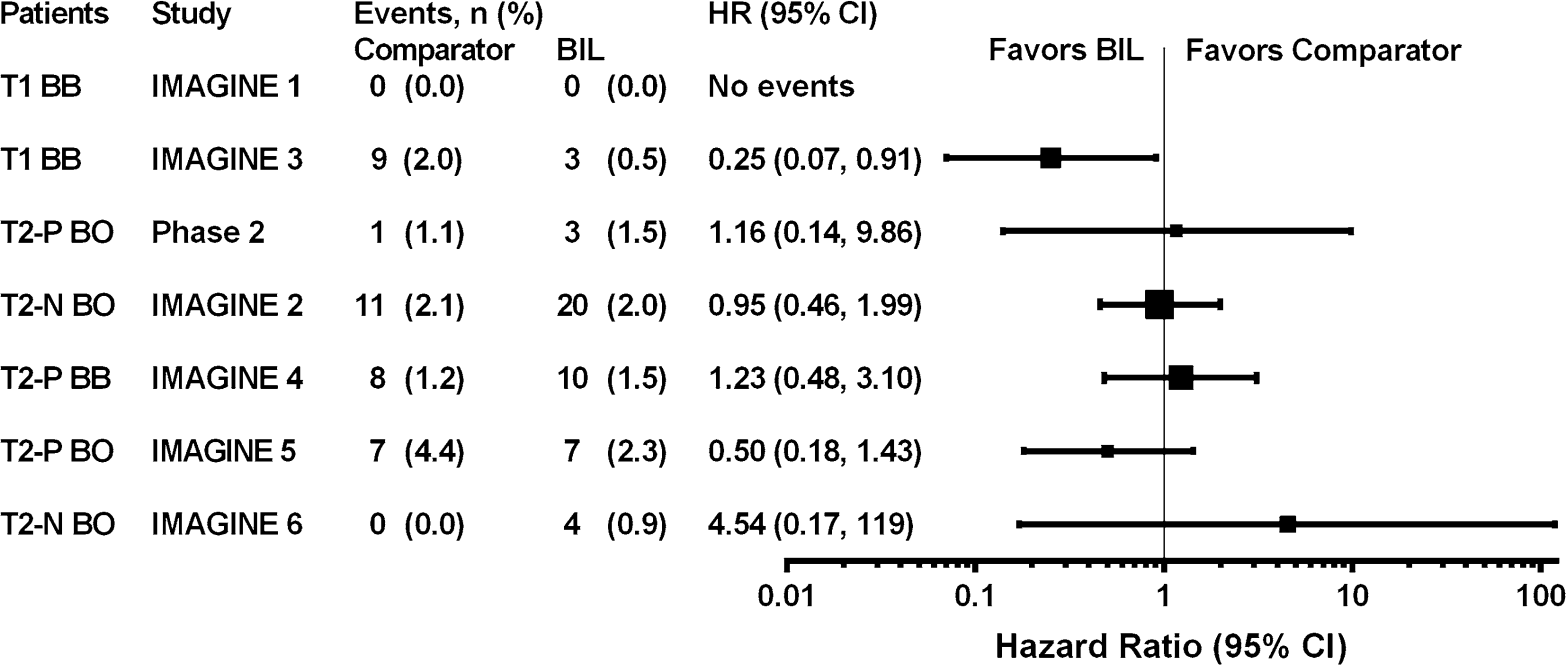
**a** Hazard ratios (HR) and 95 % CI for risk of MACE+ for treatment with BIL versus comparator insulin in the individual studies.* BB* basal-bolus insulin therapy,* BIL* basal insulin peglispro,* BO* basal only insulin therapy,* N* insulin naïve prior to study,* P* taking insulin prior to study,* T1* type 1 diabetes,* T2* type 2 diabetes

**Fig. 3:**
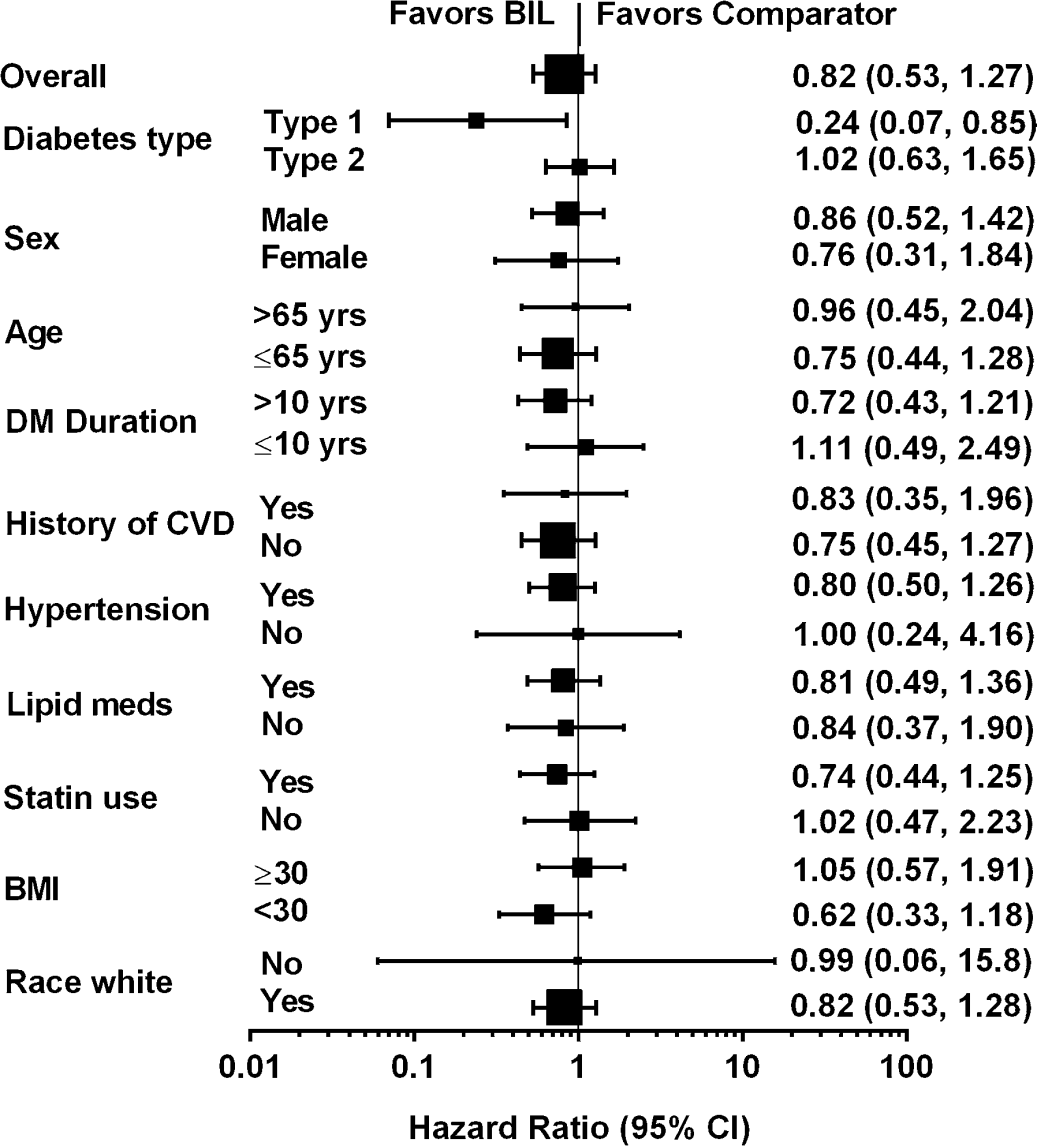
Hazard Ratios for MACE+ by subgroup. *BIL* basal insulin peglispro; *DM* diabetes mellitus; *CVD* cardiovascular disease

The results for the individual studies (Fig. 2) showed no increased risk for CVD in any patient population studied, including in type 1 diabetes and in three populations of patients with type 2 diabetes (insulin naïve with basal insulin treatment, previously on insulin with basal insulin treatment, and previously on insulin with basal-bolus treatment). Sub-group analyses, performed to look for differences in CVD risk in the overall study population by age, sex, duration of diabetes, and other characteristics, showed no evidence of increased risk with BIL versus comparator insulin in any sub-group (Fig. 3).

## Discussion

The current meta-analysis was performed to assess whether there might be any cardiovascular risk associated with a novel hepato-preferential basal insulin (based on less peripheral insulin effects) when compared to conventional insulins (glargine and NPH) in patients with type 1 or type 2 diabetes. This approach of analyzing data from phase 3 trials was consistent with that used in drug development to help inform CV risk. The current data demonstrated that there was no apparent increased MACE+ or all-cause mortality with BIL versus comparator insulin, although the upper bound of the 95 % CI in patients with type 2 diabetes did not meet the FDA guidance on oral glucose lowering agents for submission without a CV outcomes trial [33]. These analyses add to the body of data on insulin and CVD risk.

Few randomized trial data exist on the effects of insulin on CVD outcomes. DIGAMI 1 and DIGAMI 2 evaluated insulin therapy (using glucose-insulin-potassium infusions) after a myocardial infarction and the studies came to opposite conclusions (DIGAMI 1: benefit; DIGAMI 2: no benefit) [34, 35]. Heart 2D assessed the effects of prandial insulin therapy versus basal insulin on CV events and did not show a difference [36], except in a subset of patients over the age of 65 [37]. ORIGIN compared insulin glargine to standard of care in patients with impaired glucose tolerance or diabetes mellitus and did not show any difference in CVD events [3]. In the only other large data set of a basal insulin versus conventional insulin, the meta-analysis of phase 2/3 insulin degludec program reported MACE+ (current manuscript definition) with a HR of 1.097 (95 % CI: 0.681–1.768) and MACE with a HR of 1.393 (0.757–2.565) [19].

Analyses from observational data sets on possible relationships of types of insulin or total insulin dose with CV events or mortality are also limited. Three retrospective studies using database information have compared insulin types and CVD outcomes, and Siraj has performed a retrospective analysis of total insulin dose and CVD mortality [14, 38–40]. Kollhorst and colleagues analyzed 17,523 patients with type 2 diabetes from a German database who initiated NPH, a long-acting insulin analog (IG, detemir), or premixed insulins. In the primary analysis, premixed insulins were associated with higher risk for MI than long-acting analogs, but no differences were found between NPH and long-acting analogs. Propensity matched analyses showed no differences in MI among the 3 insulin groups [38]. Rhoads and associates evaluated 20,191 patients from an American national administrative claims data base (of more than 30 managed health care plans) who started NPH (N = 5461) or IG (N = 14,730). In Cox proportional hazards and Poisson regression models as well as with propensity score methods, patients treated with NPH were more likely to have an MI than those on IG [39]. Juhaeri and colleagues evaluated 65,619 patients from the PharMetrics integrated claim database who were on 4 different types of insulin regimens: (1) IG, (2) intermediate or long-acting insulins, excluding IG, (3) IG and another insulin, or (4) any other insulin regimen (rapid/short acting, premix, or 2 or more non-IG types). The authors did extensive analyses and concluded that use of IG might be associated with reduced risk for MI, but this did not reach statistical significance [40]. Whereas these data sets may be interpreted as suggesting that there may be differences among insulins with regard to CVD risk, the findings are neither consistent nor compelling. Siraj and coworkers studied the effects of insulin dose on the risk for mortality in the well-characterized cohort of patients from the ACCORD trial. Whereas there was a relationship between insulin dose and CV mortality in unadjusted models, with full adjustment for other CV risk factors, there was no relationship between total insulin dose and CV mortality [14].

In the assessment of CVD risk in patients on insulin, it is important to consider potential risk factors for CVD that may influence outcome. What follows is a discussion of potential CV risk factors that may be considered when patients treated with BIL were compared to those treated with conventional insulins. We briefly discuss variables in which there are differences between BIL and comparator insulins [triglycerides (TG), liver fat content (LFC), hypoglycemia, body weight] and try to put these changes into clinical context from observational and clinical trial data. We briefly summarize selected reports on favorable biomarker profiles with conventional insulin administration. However, there is no corresponding biomarker data in the BIL program.

In phase 2 studies of BIL in which patients had all been on insulin prior to randomization to BIL versus IG, BIL-treated patients had an increase from baseline of serum TG, while IG-treated patients did not [least squares mean (LSM) difference for type 1 diabetes: 29 mg/dL; for type 2 diabetes: 27 mg/dL] [31, 41]. In the BIL phase 3 program, patients who had previously been treated with conventional insulins and were randomized to BIL also had an increase from baseline of TG levels compared to those randomized to IG [LSM difference (BIL-IG), change from baseline to week 26 for type 1 diabetes studies: 20–24 mg/dL; for type 2 diabetes studies: 25–27 mg/dL] [25, 26, 28, 30]. However, in insulin naïve patients, those randomized to BIL had no increase in TG over the first 26 weeks of BIL treatment, while those treated with IG or NPH had a decrease in TG levels [27, 29, 42]. Thus the observed increase in TG levels appears to be predominantly the result of withdrawal from conventional insulin, although some direct effect of BIL cannot be entirely excluded. Whether TG levels contribute to CVD risk cannot be assessed from these meta-analyses.

In the BIL phase 3 program where BIL was compared to IG, patients had a mean decrease in HDL-C [LSM difference (BIL-IG), change from baseline to week 26 for type 1 diabetes studies: −1 to −3 mg/dL; for type 2 diabetes studies: 0 to −2 mg/dL]. Over the same period, patients in type 1 diabetes studies had a mean increase in LDL-C (LSM difference, change from baseline to week 26: 1–5 mg/dL), while patients in type 2 diabetes studies had a mean decrease (LSM difference, change from baseline to week 26: −1 to −5 mg/dL) [25–28, 30].

LFC at baseline was higher in patients with type 2 diabetes than in patients with type 1 diabetes [43]. In insulin naïve patients there was no increase in mean LFC in patients treated with BIL, while mean LFC declined in patients treated with IG. In patients previously treated with insulin, LFC remained unchanged with IG treatment, but increased in patients randomized to BIL [43]. Thus the observed increase in LFC appears to be predominantly the result of withdrawal from conventional insulin, although some direct effect of BIL cannot be entirely excluded. Although there are reported associations of NAFLD with CVD in diabetes patients, there are no large intervention studies demonstrating that changes in LFC are associated with changes in the CVD risk. Whether LFC contributes to CVD risk cannot be assessed from these meta-analyses.

Hypoglycemia has been associated with a risk for all-cause mortality events and CVD events [12, 44], but whether it is causally related is still uncertain. Overall, BIL was quite consistently associated with reduced risk for nocturnal hypoglycemia [45]; total hypoglycemia was comparable in patients who were treated with basal insulin only; in patients on basal-bolus insulin therapy, there was an increase in daytime hypoglycemia as well as total hypoglycemia with BIL [25, 26, 28]. As with triglycerides, the number of MACE+ was too small to do formal analyses of any association of hypoglycemia with MACE+.

In the BIL phase 3 program, patients in type 1 diabetes studies lost weight in response to BIL and gained weight in response to IG (LSM difference [BIL-IG], change from baseline to Week 26: −1.3 to −1.9 kg) [25, 26]. For patients in type 2 diabetes studies, those taking BIL gained less weight compared to those taking IG (LSM difference, change from baseline to week 26: −0.3 to −1.0 kg) [27, 28, 30]. The effects of weight change on CVD risk are still uncertain. The order of magnitude of the changes in the BIL program is unlikely to affect CV risk. This conclusion derives from the observations from the Look AHEAD study in which even greater weight differences, maintained for longer periods, were not associated with favorable effects on CV outcomes [46].

Other conventional insulins, including IG, have been associated with potentially favorable effects on biomarkers associated with CVD. For example, Chauduri and colleagues have summarized potential beneficial effects of insulin on inflammatory markers [47], Oikonomou and associates have reported increased circulating endothelial progenitor cells with IG and NPH treatment [48], and other investigators have reported favorable effects of such things as metalloproteinases and adhesion molecules [49]. However, these observations of potentially favorable effects of conventional insulins on CVD risk markers have not yet been confirmed by clinical outcomes data [47], especially comparisons among different insulins. These novel biomarkers were not evaluated in the BIL phase 2/3 program.

Whether meta-analyses of phase 3 program data are sufficient to inform CV risk can be addressed by comparing meta-analyses of phase 2/3 data from 3 glucose-lowering medications in which randomized placebo-controlled (plus standard of care) CVOTs have also been performed: SAVOR [50] (saxagliptin), EXAMINE [51] (alogliptin), and TECOS [52] (sitagliptin). Each of the development programs had done meta-analyses of the CV events from the phase 2/3 programs. The phase 2/3 meta-analysis results of MACE (41 events) with saxagliptin versus placebo in 4607 patients showed a HR of 0.45 (95 % CI: 0.24–0.83) [53], while the prospective trial (SAVOR) in 16,492 patients with a median follow up of 2.1 years reported a MACE HR of 1.00 (95 % CI: 0.89–1.12) with 1222 events [50]. The phase 2 and 3 meta-analysis of MACE with alogliptin versus placebo in 4168 patients showed a HR of 0.635 (95 % CI: 0.0–1.41) with only 23 events [54], while the HR from the EXAMINE trial in 5380 patients and a median follow up of 18 months reported a HR of 0.96 (upper bound of the 95 % CI = 1.16) with 621 MACE [51]. The phase 2 and phase 3 meta-analysis of MACE with sitagliptin versus placebo or active comparator in 14,611 patients with 78 events showed an adjusted incidence rate ratio of 0.83 (95 % CI: 0.53–1.30) [55], while the HR from the TECOS trial in 14,671 patients for MACE (the secondary composite outcome,1211 patients) was 0.99 (95 % CI: 0.89–1.11) [52].

Even though the BIL phase 2/3 program had 70 MACE and 83 MACE+ , the disparities between consistently lower hazard or risk ratios in meta-analyses than in the three CVOTs of dipeptidyl peptidase-4 inhibitors highlight the limitations of meta-analyses. The FDA guidance does suggest that both phase 2 and 3 programs select patients who are at “high” CV risk. In general, phase 3 programs include patients at low risk for CVD because of the need for monotherapy comparisons (and usually shorter duration of diabetes), renal function restrictions (such as when metformin is a comparator or background therapy), and the exclusion of patients with acute coronary syndromes or need for insulin. Patients with type 2 diabetes who require insulin therapy are generally at higher CV risk than those who require oral agents only [56]. Whether the meta-analyses of patients at higher risk for CVD based on need for insulin will be more informative than meta-analyses of programs of oral glucose-lowering drugs is uncertain.

Limitations of the meta-analyses for the BIL program beyond the general meta-analysis concerns noted above include the fact that trials of 26–78 weeks may be too short to address CVD risk. All trials had an active comparator (IG or NPH), so benefits/risks of the comparator insulins versus non-insulin therapies on CVD risk cannot be assessed. Finally, the small number of CVD events and the short duration of the phase 3 studies limit our ability to determine whether differences in putative CVD risk factors between BIL and comparator insulins (e.g., lipids, hypoglycemia, body weight, LFC) affect these observations.

Strengths of the phase 3 program include the large number of patients exposed to BIL (N = 3578) and comparator insulins (N = 2284), the fact that 3 of the trials were double-blind, and the careful identification of potential CVD events and blinded adjudication.

## Conclusions

Meta-analyses of MACE and MACE+ in patients with diabetes treated with BIL versus comparator insulins do not suggest that BIL is associated with increased CV risk. These results must be interpreted in the context of the limitations of phase 2/3 data to adequately assess CVD risk, as well as the current uncertainty as to whether differences among insulins or the total insulin dose is likely to be associated with CV risk.

## Additional file

10.1186/s12933-016-0393-6 **Table S1.** Adjudication Results. **Table S2.** Summary of MACE+ and MACE by Study in the Meta-analysis.

## Abbreviations

BIL: basal insulin peglispro
CEC: clinical events committee
CV(D): cardiovascular (disease)
IG: insulin glargine
NPH: isophane insulin
MACE: major adverse cardiac events
