# Supplementary material for: Major adverse cardiovascular events with basal insulin peglispro versus comparator insulins in patients with type 1 or type 2 diabetes: a meta-analysis
Source: Cardiovasc Diabetol. 2016 May 17;15:78. doi: 10.1186/s12933-016-0393-6 (PMC4869328; doi:10.1186/s12933-016-0393-6)
Supplement: Supplementary file 1 — 10.1186/s12933-016-0393-6 Table S1. Adjudication Results. Table S2. Summary of MACE+ and MACE by Study in the Meta-analysis. [file 12933_2016_393_MOESM1_ESM.docx]

Supplemental Data for:

Hoofwerf BJ et al. Major Adverse Cardiovascular Events with Basal Insulin Peglispro versus Comparator Insulins in Patients with Type 1 or Type 2 Diabetes: a meta-analysis

Supplemental Table 1. Adjudication Results

|  | **Total (n)** | **Adjudication Results (n)** | | | | | | | | | |
| --- | --- | --- | --- | --- | --- | --- | --- | --- | --- | --- | --- |
|  |  | **Not MACE+ or TIA, or Insuff Doc** | **CV Death** | **Non-CV Death** | **MI** | **UA** | **Stroke** | | **TIA^†^** | | **Unknown stroke type** |
| **Investigator reported events** | | | | | | | | | | | |
| Cerebrovascular event: stroke | 16 | 3 |  |  |  |  | 12 | |  | 1 | |
| Cerebrovascular event: TIA, other | 18 | 5 |  |  |  |  | 7 | | 6 |  | |
| Myocardial infarction | 35 | 8 |  |  | 27 |  |  | |  |  | |
| Unstable angina | 27 | 16 |  |  | 3 | 8 |  | |  |  | |
| Death | 29 |  | 22 | 7 |  |  |  | |  |  | |
| **Events identified by MedDRA search** | | | | | | | | | | | |
| Cerebrovascular event: stroke | 12 | 10 |  |  |  |  | 1 | 1 | |  | |
| Myocardial infarction | 112 | 106 |  |  | 3 | 3 |  |  | |  | |
| **Totals** | **249** | **148** | **101** | | | | | | | | |

^†^Transient ischemic attack is not a category of MACE or MACE+; however, this diagnosis was adjudicated to ensure that no stroke diagnosis would be missed. Insuff Doc=insufficient documentation to make a determination.

Supplemental Table 2. Summary of MACE+ and MACE by Study in the Meta-analysis

|  | | **Comparator**  **Patient years = 2016** | | | | | | **BIL**  **Patient years = 3278** | | | | |
| --- | --- | --- | --- | --- | --- | --- | --- | --- | --- | --- | --- | --- |
| **Patient Group** | **Study/ Weeks** | **Comp** | **N** | **MACE+ n (%)** | **Rate^†^** | **MACE**  **n (%)** | **Rate^†^** | **N** | **MACE+ n (%)** | **Rate^†^** | **MACE**  **n (%)** | **Rate^†^** |
| T1D | IMAGINE 1/78 | GL | 159 | 0 (0.0) | 0.0 | 0 (0.0) | 0.0 | 294 | 0 (0.0) | 0.0 | 0 (0.0) | 0.0 |
|  | IMAGINE 3/52 | GL | 449 | 9 (2.0) | 2.0 | 8 (1.8) | 1.8 | 663 | 3 (0.5) | 0.5* | 3 (0.5) | 0.5 |
| T2D  Basal-bolus | IMAGINE 4/26 | GL | 677 | 8 (1.2) | 2.1 | 6 (0.9) | 1.6 | 691 | 10 (1.5) | 2.6 | 7 (1.0) | 1.8 |
| T2D  Basal only | Phase 2/12 | GL | 93 | 1 (1.1) | 3.5 | 1 (1.1) | 3.5 | 195 | 3 (1.5) | 5.3 | 1 (0.5) | 1.8 |
|  | IMAGINE 2/52-78 | GL | 535 | 11 (2.1) | 1.7 | 8 (1.5) | 1.2 | 1003 | 20 (2.0) | 1.6 | 19 (1.9) | 1.5 |
|  | IMAGINE 5/52 | GL | 159 | 7 (4.4) | 4.5 | 7 (4.4) | 4.5 | 305 | 7 (2.3) | 2.3 | 7 (2.3) | 2.3 |
|  | IMAGINE 6/26 | NPH | 212 | 0 (0.0) | 0.0 | 0 (0.0) | 0.0 | 427 | 4 (0.9) | 1.7 | 3 (0.7) | 1.3 |
| Total |  |  | **2284** | **36 (1.6)** | **1.8** | **30 (1.3)** | **1.5** | **3578** | **47 (1.3)** | **1.4** | **40 (1.1)** | **1.2** |

**^†^**MACE+ or MACE events/100 patient-years; *p<0.05 for between group comparison; comp=comparator; GL, insulin glargine; NPH, isophane insulin.
